# An SVM-Based Classifier for Estimating the State of Various Rotating Components in Agro-Industrial Machinery with a Vibration Signal Acquired from a Single Point on the Machine Chassis

**DOI:** 10.3390/s141120713

**Published:** 2014-11-03

**Authors:** Ruben Ruiz-Gonzalez, Jaime Gomez-Gil, Francisco Javier Gomez-Gil, Víctor Martínez-Martínez

**Affiliations:** 1 Department of Signal Theory, Communications and Telematics Engineering, University of Valladolid, Valladolid 47011, Spain; E-Mails: jgomez@tel.uva.es (J.G.-G.); vmarmar@ribera.tel.uva.es (V.M.-M.); 2 Department of Electromechanical Engineering, University of Burgos, Burgos 09006, Spain; E-Mail: fjggil@ubu.es

**Keywords:** Support Vector Machine (SVM), predictive maintenance (PdM), agricultural machinery, condition monitoring, fault diagnosis, vibration analysis, feature extraction and selection, pattern recognition

## Abstract

The goal of this article is to assess the feasibility of estimating the state of various rotating components in agro-industrial machinery by employing just one vibration signal acquired from a single point on the machine chassis. To do so, a Support Vector Machine (SVM)-based system is employed. Experimental tests evaluated this system by acquiring vibration data from a single point of an agricultural harvester, while varying several of its working conditions. The whole process included two major steps. Initially, the vibration data were preprocessed through twelve feature extraction algorithms, after which the *Exhaustive Search* method selected the most suitable features. Secondly, the SVM-based system accuracy was evaluated by using *Leave-One-Out* cross-validation, with the selected features as the input data. The results of this study provide evidence that (i) accurate estimation of the status of various rotating components in agro-industrial machinery is possible by processing the vibration signal acquired from a single point on the machine structure; (ii) the vibration signal can be acquired with a uniaxial accelerometer, the orientation of which does not significantly affect the classification accuracy; and, (iii) when using an SVM classifier, an 85% mean cross-validation accuracy can be reached, which only requires a maximum of seven features as its input, and no significant improvements are noted between the use of either nonlinear or linear kernels.

## 1. Introduction

Agro-industrial machinery has a high initial investment and requires regular maintenance if further expensive repairs are to be avoided [1]. The need for machine maintenance programs and their appropriateness is a well-argued topic in the industry, which is reflected in the literature [2]. Early detection of a mechanical component that is malfunctioning will lead to its prompt replacement, thereby avoiding more costly repairs in the future.

Nowadays, many predictive maintenance techniques are employed, in order to reduce hazards and subsequent failures of machinery [1–3]. According to Scheffer *et al.* [4], the main such techniques are vibration monitoring, acoustic emission, oil analysis, particle analysis, corrosion monitoring, thermography, and performance monitoring.

Vibration analysis is a non-intrusive method that is widely employed in machinery status inspections, mainly on rotating equipment including engines, turbines, and compressors, among others [4–6]. In the case of machinery with no vibration isolation, the vibration signal propagates throughout the whole structure of the machine with moderate attenuation. The propagation of these vibrations makes it possible to monitor certain rotating components by placing an accelerometer at a different point on the machine structure [7]. However, the propagation of vibrations has a disadvantage, in so far that it transmits various vibration signals from various other machine components, in addition to the signal of interest, making it more difficult to identify the relevant signal [8].

Vibration signals from rotating components are usually analyzed in the frequency domain, because significant peaks in the signal spectrum appear at frequencies that are related to the rotation frequency of the component [4]. Various authors have performed this analysis using fast Fourier transform [9], short-time Fourier transform [10], the wavelet transform [11–15], the S-transform [16], and the Hilbert-Huang transform [17–20], among others. Due to the relationship between the rotation frequency of the machine component and the highest peaks in the spectrum signal, experts can estimate the status of machine components by looking for patterns in the spectrum signal. Nevertheless, doing so requires expert analysis of the vibration signal spectrum, which implies detailed knowledge of the machine, the way it functions and full information on the rotation speed of the component. Automated systems have been proposed to estimate the status of machine components using frequency analysis in the absence of expert analysis [19, 21]. These systems incorporate knowledge of the machine component to extract characteristics from the spectrum signal and to estimate its status on the basis of these characteristics.

A Support Vector Machine (SVM) [22] is a supervised learning model widely used in the discipline of pattern recognition for classifying purposes. Due to its learning and generalization capabilities it is well suited for the implementation of estimation methods, which are widely required in automated diagnosis systems. According to the literature, many SVM-based applications have successfully been implemented [23], both for classification [24] and nonlinear regression [25]. Numerous improvements have been proposed over recent years that focus specifically on vibration monitoring in machinery fault diagnosis [26, 27]. Widodo and Yang [28] offered a very thorough review of the latest major advances in the field of SVM-based vibration analysis for predictive maintenance.

Although a good deal of research has previously examined SVMs in machinery predictive maintenance [21, 28], to the best of our knowledge, automatic prediction of the state of various rotating components in an agro-industrial machine by employing only one vibration signal acquired from a single point on the machine chassis, has not been conducted in previous research.

The purpose of this article is to present evidence to assess the feasibility of estimating the state of various rotating components in agro-industrial machinery by employing one vibration signal acquired from a single point on the machine chassis as the system input. The following five rotating component states in an agricultural harvester were selected to assess that estimation capability: (1) engine speed status (*high speed*/*low speed*); (2) threshing cylinder operating status (*on*/*off*); (3) threshing cylinder balance status (*balanced*/*unbalanced*); (4) straw chopper operating status (*on*/*off*); and (5) straw chopper balance status (*balanced*/*unbalanced*).

## 2. Background

This section comprises some fundamentals about vibration analysis in agro-industrial machinery, classification in supervised machine learning, feature extraction and selection, and SVM-based classification.

### 2.1. Vibrations in Agro-Industrial Machinery

Vibration can be defined as the repeated motion of a certain component back and forth from a given position. Accelerometers are sensors that measure proper acceleration. These devices are the most widely used for capturing vibration signals in rotating machinery applications. Typical accelerometers capture signals in frequency ranges from 1 Hz to 10 kHz [4].

The most common defects causing high vibration levels in machinery, in accordance with Scheffer *et al.* [4], are: unbalance of rotating parts, misalignment of couplings and bearings, bent shafts, worn or damaged gears and bearings, bad drive belts and chains, torque variations, electromagnetic forces, aerodynamic forces, hydraulic forces, looseness, rubbing, and resonance. When machinery rotating components operate at high speeds or under harsh operating conditions for a long time, some of these defects start to appear.

Vibrations can reveal the presence of machinery defects. Usually, vibrations on rotating components appear at specific frequencies, which are characteristic of each specific component and also depend on the component rotation speed and other properties [19, 21]. Traditionally, depending on the vibration amplitude at those specific frequencies, the severity of the defects can be assessed. Therefore, plenty of information on the condition of a component, e.g., possible deterioration, can be detected by analyzing the vibration characteristics of isolated components [4].

In addition, the vibration signals of a specific machine component can be acquired from almost any point on the machine structure, even though the signals will be slightly attenuated, due to their propagation throughout the machine structure and the imperfect isolation of the main sources of vibration. Propagation complicates data processing and the extraction of useful information, because information from several machine components is mixed. It nevertheless greatly simplifies the data acquisition stage, as just a sensor may be installed at a single point on the machine.

### 2.2. Classification in Supervised Machine Learning

Machine learning, as a sub-field of artificial intelligence in computer science, deals with intelligent systems that can modify their behavior in accordance with the input data. Intelligent systems must have the capability of deducing the function that best fits the input data, in order to learn from the data. Machine learning can be divided into unsupervised and supervised learning, depending on the information that is available for the learning process. Unsupervised machine learning undertakes the inference process by using an unlabeled training set, *i.e.*, without any information on the desired output, and it seeks to deduce relationships by looking for similarities in the dataset. Meanwhile, supervised machine learning assumes that a labeled training set, for which the desired output is completely known, is available.

Classification, as a branch of supervised learning, is defined as the process of identifying the class to which a previously unseen observation belongs, based on previous knowledge given by a training dataset that contains instances the category membership of which is certain. Any algorithm which performs classification tasks, *i.e.*, the mapping of input data to an assigned class, is called a classifier.

Classifiers must be trained, based on previous knowledge, in order to function properly. The training process makes use of a sample of *N* observations, the corresponding classes of which are certain. This sample of *N* observations is typically divided into two subsamples: the training and the test datasets. Firstly, the training dataset is used in the process of computing a classifier that is well-adapted to these data. Then the test dataset is used to assess the generalization capability of the previously computed classifier.

Both the misclassification rate and the success rate in the test dataset are commonly used as quality measurements to assess classifier performance. The misclassification rate is defined as the proportion of observations which are wrongly assigned to an incorrect class. It is expressed as follows:

$$MR=\frac{\text{Number of Incorrect Classifications}}{\text{Total Number of Classifications}}$$

Alternatively, the success rate (also called the hit rate) is defined as the proportion of observations that are properly assigned to the corresponding class and is calculated as follows:

$$SR=\frac{\text{Number of Correct Classifications}}{\text{Total Number of Classifications}}=1-MR$$

The *k-fold cross-validation* is an enhanced method of evaluating classifier performance, especially with small training and test datasets. In this method, the original sample of *N* observations is randomly partitioned into *k* subsamples of equal size. From those *k* subsamples, a single subsample is retained as the test dataset, and the remaining *k* – 1 subsamples are used as the training dataset. The *k-fold cross-validation* repeats this training and test process *k* times, using each of the *k* subsamples only once as the test dataset. Cross-validation accuracy is calculated as the average of the success rate obtained for each of the *k* different test datasets. When *k* = *N*, *k*-fold cross-validation is also known as *leave-one-out cross-validation*.

Many different classifiers have been proposed in the literature [29, 30]. Some of the main ones include k-nearest neighbor classifier, Bayes classifier, logistic regression, Fisher's linear discriminant, decision tree, Artificial Neural Networks (ANN), and Support Vector Machines (SVM). An SVM classifier is used in this article and hence SVM is described in greater detail in Section 2.4.

### 2.3. Feature Extraction and Selection for Classification

Machine learning systems, including classifiers, are typically required to process large volumes of information. The application of dimensionality reduction techniques to the input data prevents the classifier from processing too much data and improves its performance. Dimensionality reduction, within statistical machine learning field, is defined as the process of reducing the number of variables of a dataset while retaining most of its degrees of freedom, thereby simplifying the subsequent classification problem. Feature extraction and selection are methods to accomplish dimensionality reduction.

Feature extraction [29] consists in reducing the dimensions of a *d*-dimensional input data vector by transforming it into a new *m*-dimensional output data vector, where *m* < *d*. The resulting *m*-dimensional vector, called feature vector, should retain from the original vector most of the useful information for the subsequent classification stage. This property is often referred as *degrees of freedom preservation*. Attending to their data type, features can be categorical, ordinal, integer-valued, or real-valued. A very wide variety of feature extraction algorithms have been proposed in the literature [31–34]. A taxonomy of these algorithms exists on the basis of their relationship to specific mathematical fields. The most popular such categories are nonlinear, statistical and transformed-domain based. Some of the nonlinear feature extraction algorithms are *Correlation Dimension* [35], *Kolmogorov Complexity* [36], *Lempel-Ziv Complexity* [31, 37], *Approximate Entropy* [38], and *Sample Entropy* [39]. Classical time-domain based methods of statistical feature extraction include *Mean Value*, *Standard Deviation*, *Skewness*, *Kurtosis*, *Average Power*, and *Shannon Entropy* [40]. Some of the notable frequency domain feature extraction techniques are *Spectral Entropy* [32], *Median Frequency* [33, 41], *Bandwidth Containing 90% of the Signal Energy*, and *Relative Wavelet Packet Energy* [34].

Feature selection [29] involves choosing, among an original set of features of size *m*, the subset of size *n* that best represents the original set and that yields the smallest classification error. The feature selection process can be conducted, among other methods by means of *Exhaustive Search* or *Sequential Forward/Backward Floating Search* [29]. On the one hand, *Exhaustive Search* explores all the possible subsets, *i.e.*, 2*^m^* if *n* is a free parameter, or $\left(\begin{array}{c} m\\ n \end{array}\right)$ if *n* is a preset constant. This method therefore guarantees the selection of the best subset, although its use of computational resources is excessive. On the other hand, *Sequential Forward/Backward Floating Search* restricts the search to a smaller subtree by only allowing feature deletion and addition at each step. Consequently, this method presents a more affordable computational load, but it fails to guarantee optimal subset selection, even though it has been proven to yield suboptimal results that are almost optimal.

The performance improvements offered by feature extraction and selection techniques are linked to: (i) dimension reduction that mitigates the ‘curse of dimensionality’ problem and therefore reduces the risk of over-fitting [29, 42]; and (ii) simplification of the resulting classifier, which results in using less memory and fewer computational resources [29].

### 2.4. Support Vector Machines for Classification

*Support Vector Machines* (SVM) is a statistical supervised machine learning technique, used both for classification and for regression purposes. Originally proposed by Vapnik and Cortes [22, 43], in 1995, although its principles and derivation differ from those of *Artificial Neural Networks* (ANN), some authors sometimes consider SVMs as a special kind of ANN [44]. However, many authors refuse to do so, due to essential differences between SVM and ANN techniques [45]. While SVM mechanisms are mainly based on a rigorous geometrical and statistical approach, ANNs try to emulate the behavior of the human brain and its neural system.

The original SVM proposal was aimed at both the binary classification problem, considering only two possible classification classes, and the multiclass classification problem, which considers more than two classification classes.

Binary linear SVM classification performs the calculation of the optimal hyperplane decision boundary, separating one class from the other, on the basis of a training dataset. Optimality can be understood, depending on whether perfect classification of the training dataset is feasible and desired, in two separate ways:

- If perfect separability of training dataset classes can be achieved, a Hard Margin optimality can be used. In this case, the hyperplane decision boundary is chosen to maximize the distance from the hyperplane to the nearest training data point.
- If perfect classification is not desired or if it is impossible, a Soft Margin optimality is used. In this case, the hyperplane selection is a customizable tradeoff between minimizing the misclassification rate and maximizing the distance to the nearest properly classified training point.

**Figure 1.**
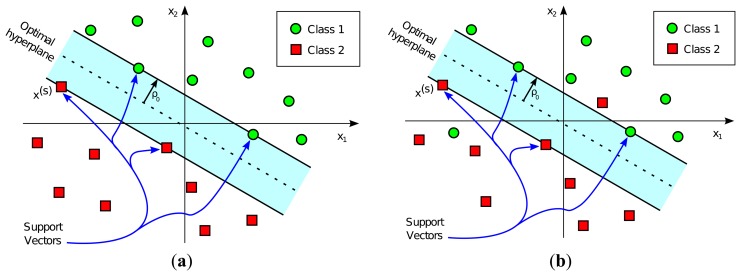
Representation of a Support Vector Machine (SVM)) classifier corresponding to (**a**) a linearly separable pattern, where the hyperplane totally separates green circles from red squares; and (**b**) a non-linearly separable pattern, where no hyperplane separates all the green circles from the red squares.

The decision boundary hyperplane in SVM classification is calculated by employing the training dataset. This decision boundary is completely determined by the so-called Support Vectors, a subset of training input vectors which by themselves alone lead to the same decision boundary. After this hyperplane is determined, the SVM classifier is ready to be used with a different dataset from the one used in the training stage. The assigned class, labeled either +1 or −1, depends on the side of the decision boundary on which the input vector falls. Figure 1 represents a graphical example of linear SVM-based classification, both in the case of linearly separable classes and non-linearly separable classes.

SVM multiclass classification usually tackles the classification and computation of the decision boundary by reducing the problem to a set of binary classification problems. The main such approaches are *pairwise* and *one-versus-all* classification methods [46]. Compact multiclass reformulations of the binary classification problem have also been proposed [46].

To be mathematically rigorous, the most general SVM linear binary classification problem can be stated as follows:

“*Given a training dataset*, ${\left\{x_{i},d_{i}\right\}}_{i=1}^{N}$, *the goal is to compute the optimal weight vector* ***w***, *the goal is to compute the optimal weight vector* ***ξ***, *such that satisfy the following constraints:*

$$\begin{array}{c} d_{i}\left(w^{T}x_{i}+b\right)\geq 1-\xi_{i},\;\forall i=1,\;2,\ldots ,N\\ \xi_{i}\geq 0,\;\forall i=1,\;2,\ldots ,N \end{array}$$

*and such that the following cost function is minimized:*

$$\Phi \left(w,\xi \right)=\frac{1}{2}w^{T}w\;+C\sum_{i=1}^{N}\xi_{i}$$

*where*, ***x****_i_* ∈ ℝ^*m*_0_^ *denotes the i-th input vector, d_i_* ∈ {–1, 1} *denotes the class corresponding to the i-th input vector*, $\xi ={\left\{\xi_{i}\right\}}_{i=1}^{N}$ *represents the slack variables, and the constant C is a user-specified parameter that determines the tradeoff between misclassification and maximum inter-class margin.”*

**Figure 2.**
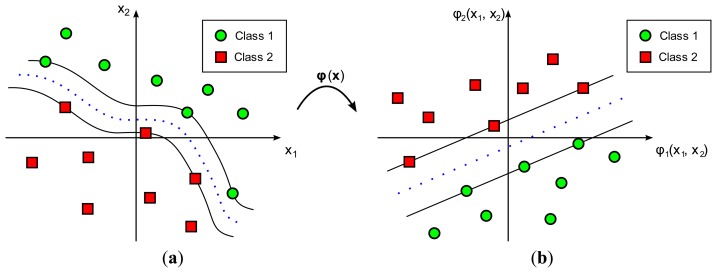
Representation of a Support Vector Machine classifier with a nonlinear kernel. Function ***φ***(·) is the nonlinear transformation mapping vectors from (**a**) the input space to (**b**) the feature space.

In practice, most classification problems cannot be solved by using a simple hyperplane as the decision boundary. In such cases a more complex and elaborate decision boundary is required. SVM achieves this goal by increasing the dimensionality of the input space, of dimension *m*_0_, by applying a nonlinear transformation, denoted by ***φ***(·), into a feature space of dimension *m_f_* > *m*_0_ (Figure 2). This transformation, ***φ***(·), serves to reduce the misclassification probability in the transformed feature space. The most typical transformation functions, as in the case of ANNs, are *radial basis functions*, *higher-order polynomials*, and *sigmoids*. Figure 2 represents a graphical example of an SVM nonlinear classification.

The boundary in the nonlinear classification problem is still a hyperplane, not in the original input space but in the feature space, and can be expressed as the points ***φ***(***x***) that satisfy that:

(1) $$w^{T}\varphi (x)+b=0$$

where, ***x*** ∈ ℝ^*m*_*0*_^ and ***φ***(***x***) ∈ ℝ*^m_f_^*.

Following the application of the Lagrange multipliers method, it has been shown that the optimal weight vector can be expressed as [44]:

(2) $$w=\sum_{i=1}^{N}\alpha_{i}d_{i}\varphi (x_{i})$$

where, *α_i_* stands for the Lagrange multiplier coefficients.

Therefore, the optimal decision boundary can be rewritten as:

(3) $$\sum_{i=1}^{N}\alpha_{i}d_{i}\varphi {\left(x_{i}\right)}^{T}\varphi (x)+b=0$$

Renaming *u_i_* = *α_i_d_i_* and *K*(***x****_i_*, ***x***) = ***φ***(***x****_i_*)*^T^* ***φ***(***x***) = ***φ***(***x***)*^T^* ***φ***(***x****_i_*) = *K*(***x***, ***x****_i_*), the decision function, *y*, can be expressed as:

(3) $$y=\sum_{\text{i}=1}^{N}u_{i}K\left(x,x_{i}\right)+b$$

In case of linear classifiers, *K*(***x***, ***x****_i_*) is the conventional Euclidean inner product of the input vector ***x*** with the Support Vector ***x****_i_*. In case of nonlinear classifiers, *K*(***x***, ***x****_i_*) is the conventional Euclidean inner product of the nonlinear transformation ***φ***(***x***) of the input vector ***x*** with the nonlinear transformation ***φ***(***x****_i_*) of the Support Vector ***x****_i_*.

**Figure 3.**
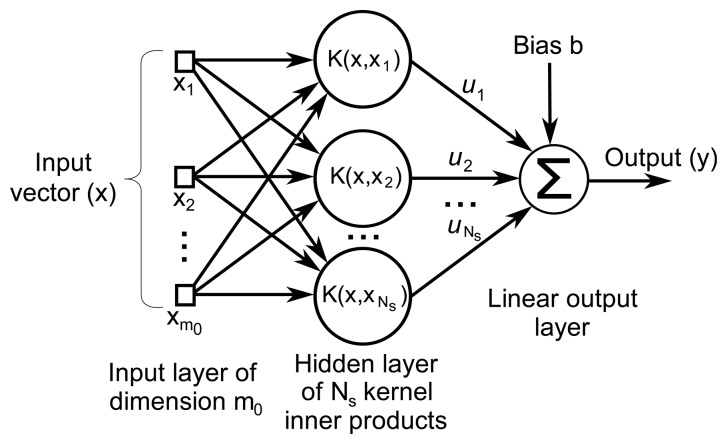
Architecture of a Support Vector Machine classifier. Inner product kernels, K(·, ·), denote the *m*_0_-dimensional kernel inner product of the input vector with each of the *N_s_* Support Vectors.

The decision function in Equation (4) results in the architecture depicted in Figure 3, once the proper weights and Support Vectors have been computed in the training stage. Only the Support Vectors have to be considered, as they are the only vectors that generate non-zero *α_i_* coefficients [44].

Classification is therefore performed by identifying the sign of the output value, *y*, in Equation (4). If *sign*(*y*) = +1, then this input is labeled as class +1 and if otherwise as class −1.

The most well-known and widely used nonlinear kernels are *radial basis functions* (*RBF*), *sigmoids*, and *polynomials*. The *RBF kernel* can be expressed as *K*(***x***, ***y***) = *exp*(–γ ‖***x***–***y***‖^2^), where γ is a user-defined parameter; the *sigmoidal kernel* can be expressed as *K*(***x***, ***y***) = *tanh*(*γ* ***x*^T^*y***), where γ > 0 and *c*_0_ < 0 are user-defined parameters; and, the *d-order polynomial kernel* can be expressed as *K*(***x***, ***y***) = *tanh*(*γ* ***x*^T^*y* +** *c*_0_)*^d^*, where γ and *c*_0_ are user-defined parameters and where *d* denotes the polynomial degree. Other kernels may also be found, in addition to those listed above.

The underlying SVM training process undertakes the problem of minimizing a quadratic functional subject to linear constraints. This problem, known as *Quadratic Programming*, has a closed solution. Although the solution can be analytically computed by applying the Lagrange multipliers method, other computational methods are typically used, especially when the dimensionality of the problem becomes high. Some of these methods include, among others, *Interior Point* methods [47], the *Sequential Minimal Optimization* (SMO) algorithm [48, 49], *Incremental* methods [50], and the *Kernel-Adatron* (KA) algorithm [51]. More information about the SVM training process has been gathered by Campbell and Ying [52].

Those readers eager to discover the rigorous mathematical statement and solution of the problem underlying Support Vector Machines are encouraged to read the comprehensive introduction to SVM provided by Haykin [44] or the in-depth work by Steinwart and Christmann [53].

## 3. Materials and Methods

**Figure 4.**
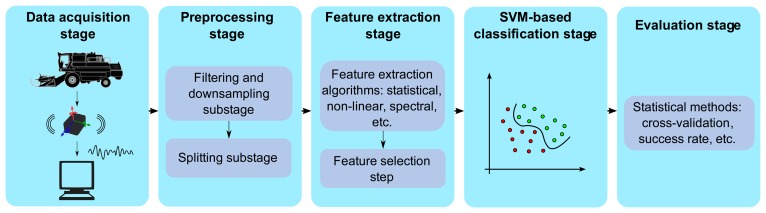
Overall block diagram summarizing the main processing stages.

The main processing stages performed in this study can be conceptualized as follows: (i) the data acquisition stage (Section 3.1); (ii) the preprocessing stage (Section 3.2); (iii) the feature extraction and selection stage (Section 3.3); (iv) the SVM-based classification stage (Section 3.4); and (v) the evaluation stage (Section 3.5). Figure 4 summarizes the main processing stages and contains a high-level description of the methods, which are explained in greater detail in the remainder of this section.

### 3.1. Data Acquisition Stage

**Figure 5.**
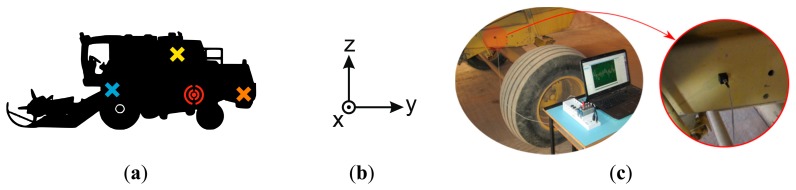
(**a**) Harvester schematic in which the red symbol represents the precise location of the accelerometer sensor on the chassis, the yellow cross represents the location of the engine, the blue cross represents the location of the threshing cylinder, and the orange cross represents the location of the straw chopper; (**b**) The coordinate axes of the accelerometer in this study were as follows: the *x* axis was transverse to the front direction of the harvester, the *y* axis pointed to the reverse direction of the harvester, and the *z* axis was upward vertical with respect to the ground; (**c**) The experimental setup for data acquisition and a close up of the position of the Kistler 8690C50 triaxial accelerometer.

Vibration data were experimentally obtained from an eleven-year-old New Holland TC56 harvester that had clocked 3800 working hours. Vibration signals were acquired from a stationary harvester operating in threshing mode. A Kistler 8690C50 triaxial accelerometer was used to measure the vibration signals on transverse, longitudinal and vertical axes (Figure 5). After several trial and error tests, the accelerometer sensor was placed on the left hand side of the harvester chassis, neither very close nor very far away from the rotating components under analysis (Figure 5). The sensor was positioned by using an adhesive mounting following the guidelines in Scheffer *et al.* [4]. This mounting method was selected because the frequency analysis in this article was bandlimited below 200 Hz and it permits accurate measurements within this frequency range [4]. Vibration signals were acquired using the *NI Sound and Vibration Assistant* software and a *National Instruments* (NI) data acquisition (DAQ) system. The data acquisition system was composed of an *NI 9234* data acquisition module for analog input signals and an NI compact DAQ chassis *NI cDAQ-9172*, to connect the DAQ module to a laptop.

A total of 18 different data acquisition processes were performed to acquire data on all the combinations of the following harvester working conditions: (i) engine speed status (*high speed*/*low speed*); (ii) threshing cylinder operating status (*on*/*off*); (iii) threshing cylinder balance status (*balanced*/*unbalanced*) in the *on* operating status; (iv) straw chopper operating status (*on*/*off*); and (v) straw chopper balance status (*balanced*/*unbalanced*) in the *on* operating status. The straw chopper was unbalanced on purpose by the breakage of a blade. The unbalance was provoked in this way, because blade breakage against stones is a frequent cause of unbalances. The threshing cylinder was unbalanced by adding an eccentric weight to it. The unbalance was provoked in this way, because the threshing cylinder can typically become unbalanced when its bars suffer from non-uniform wear, due to usage and an eccentric weight simulates the same effect.

Sixty-second long epochs or frames of machine operation were recorded for each of the 18 acquisition processes, using a sampling frequency of 1706.48 Hz, which generated a total of 99, 120 samples per epoch.

### 3.2. Preprocessing Stage

**Figure 6.**
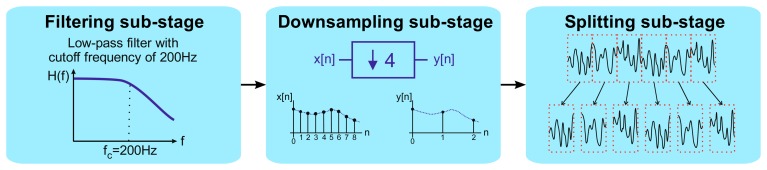
Block diagram representing the three preprocessing sub-stages.

The acquired acceleration time-series data were first preprocessed in order to adapt them to the subsequent feature extraction stage. The entire preprocessing stage was divided into the following three sub-stages (Figure 6): (*i*) a low-pass filtering sub-stage; (*ii*) a downsampling sub-stage; and (*iii*) a splitting sub-stage.

In the first sub-stage, low-pass filtering took place. A digital IIR elliptic low-pass filter, with a cutoff frequency of 200 Hz, was applied to the input signal. The vibration frequencies of interest, which are the main harmonics of the components rotation speeds, are located within the range from 0 to 200 Hz. This filtering was performed in order to remove noise and unwanted interferences to achieve a better performance.

Next, after filtering, the downsampling sub-stage took place. The input signal was decimated, in order to reduce the sampling frequency by a factor of *N_fs_*, where *N_fs_* ∈ ℕ is the decimation ratio. In this article, a value of *N_fs_* = 4 was chosen, taking into account the frequency range of interest. Therefore, the effective sampling frequency was reduced after downsampling from 1706.5 Hz, the one originally employed in the acquisition stage, to 426.625 Hz.

Finally, the splitting sub-stage was conducted. The downsampled signal, coming from the second sub-stage, was then split into six epochs of about ten seconds, which was the frame size considered sufficient for keeping meaningful information on the vibration signal for the posterior feature extraction steps and for ensuring good frequency resolution in the subsequent FFT analysis. In this way, a total of 4130 samples per epoch were obtained.

All these preprocessing tasks were performed with *MATLAB*^®^ program.

### 3.3. Feature Extraction and Selection Stage

This stage involves the dimensionality reduction of the input data-series coming from the previous stage. It is divided into two sub-stages: feature extraction and feature selection.

Firstly, the preprocessed data from the previous stage were brought in the feature extraction sub-stage, in order to achieve a simpler classifier. Then, the input signal for this stage, denoted as *x*[*n*], where *n* = 1, 2, …, *N* = 4130, was used to extract the following features:

(1) *Average Power (P)*, defined as $\bar{P}=\frac{1}{N}\sum_{n=1}^{N}x{\left[n\right]}^{2}$. This feature quantifies the overall vibration intensity.
(2) *Sample Entropy (SampEn)*, computed using the definition provided by Richman *et al.* [39]. This feature is a measurement of signal regularity that assigns higher values to more random data; for instance when multiple vibration sources are superposed.
(3) *Spectral Entropy (SpecEn)*, computed in the same way as by Hornero *et al.* [33]. This feature was employed because of its capability to quantify the flatness of the spectrum. The more frequency peaks the signal has, the greater this feature becomes.
(4) *Mean Value (x̄)*, calculated as $\bar{x}=\frac{1}{N}\sum_{n=1}^{N}x\left[n\right]$. It reflects the amplitude of low frequency background vibrations.
(5) *Median frequency (MF)*, computed as the frequency which divides the power spectrum into two halves, each of which contains the same energy. It was calculated in the same way as by Hornero *et al.* [33].
(6) *Standard Deviation (σ)*, calculated by using the mean value *x̄* that has previously been defined, as the square root of the unbiased estimator of the variance, *i.e.*, $\sigma =\sqrt{\frac{1}{N-1}\sum_{n=1}^{N}{\left(x\left[n\right]-\bar{x}\right)}^{2}}$. This feature provides information on the width of the amplitude histogram distribution, supplying additional information on the shape of the vibration signal.
(7) *Skewness ( s*_0_ *)*, calculated as the unbiased estimator $s_{0}=\frac{\sqrt{N\left(N-1\right)}}{N-2}\frac{\frac{1}{N}\sum_{N}^{n=1}{\left(x\left[n\right]-\bar{x}\right)}^{3}}{{\left(\sqrt{\frac{1}{N}\sum_{N}^{n=1}{\left(x\left[n\right]-\bar{x}\right)}^{2}}\right)}^{3}}$ Skewness, which is a measure of histogram distribution asymmetry around its mean, can reflect vibration asymmetries due to mechanical faults.
(8) *Kurtosis (k*_0_*)*, calculated as the unbiased estimator $k_{0}=\frac{N-1}{\left(N-2\right)\left(N-3\right)}\left(\left(N+1\right)k_{1}-3\left(N-1\right)\right)+3$, where $k_{1}=\frac{\frac{1}{N}\sum_{N}^{n=1}{\left(x\left[n\right]-\bar{x}\right)}^{4}}{{\left(\frac{1}{N}\sum_{N}^{n=1}{\left(x\left[n\right]-\bar{x}\right)}^{2}\right)}^{2}}$. This feature reflects the peakedness of the histogram, giving information on the distribution of the vibrations amplitude.
(9) *Central Tendency Measurement (CTM)*. In the first place, the first-order differences scatter plot is constructed, representing *x*[*n*+1]–*x*[*n*] on the *X* axis against *x*[*n*+2]–*x*[*n*+1] on the *Y* axis. The proportion of points lying inside a circle of a certain fixed radius is then returned as a measurement of signal regularity. A radius of 0.05 g was selected in this study, which is an appropriate one for distinguishing the related classification classes. This feature offers a measurement of the randomness of the vibration signal, where a low value of this feature implies sharp changes in the vibration signal. Sharp changes in vibration signals may be caused both by high frequency vibrations or sudden transitions due to mechanical faults.
(10) *Correlation coefficient (r) from the first-order differences scatter plot*. As with the previous feature, the first-order differences scatter plot is constructed first, obtaining both *X*[*n*] and *Y*[*n*] vectors. Then the Pearson's linear correlation coefficient between both vectors is computed as: $r=\frac{\sum_{N-2}^{n=1}\left(X\left[n\right]-\bar{X}\right)\left(Y\left[n\right]-\bar{Y}\right)}{\sqrt{\sum_{N-2}^{n=1}{\left(X\left[n\right]-\bar{X}\right)}^{2}}\sqrt{\sum_{N-2}^{n=1}{\left(Y\left[n\right]-\bar{Y}\right)}^{2}}}$, where *X̄* and *Ȳ* are the mean values of *X*[*n*] and *Y*[*n*], respectively. This feature offers a measurement of the unpredictability of the signal from the previous data; the higher the measure of |*r*| the more predictable the signal is.
(11) *Lempel-Ziv Complexity (LZC)*, computed as by Hornero *et al.* [33]. This feature offers a notion of complexity in a statistical sense. It characterizes the average information quantity within a signal and can therefore reflect the superposition of several vibration sources.
(12) *Crest Factor (C)*, calculated as $C=\frac{\underset{n}{\max}\left|x\left[n\right]\right|}{\sqrt{\frac{1}{N}\sum_{i}x{\left[n\right]}^{2}}}$, where *N* is the number of samples of the time-series *x*[*n*]. This feature reflects the spikiness of the signal with respect to its RMS value and is therefore useful to assess the presence of mechanical faults.

All of the above algorithms were selected on the basis of the previous literature on vibration analysis [21, 54–56] and by extrapolating ideas from studies in other fields [33, 37, 39].

Secondly, after having extracted these features from the preprocessed data-series, the most suitable features from among them all were selected in the feature selection sub-stage. The feature selection process was undertaken by using the *Exhaustive Search* method, which explores all of the possible feature subsets. With each of the explored subsets, linear SVM *leave-one-out* cross-validation was performed to assess the goodness of this subset. The feature subset with highest cross-validation accuracy was selected. The value of parameter *C*, involved in the SVM classification problem, was prefixed at 1 in all cases. Cross-validation accuracy was calculated for each classifier undertaking each of the five classification problems, corresponding to the five rotating component states of the agricultural harvester under consideration: (1) engine speed status (*high speed*/*low speed*); (2) threshing cylinder operating status (*on*/*off*); (3) threshing cylinder balance status (*balanced*/*unbalanced*); (4) straw chopper operating status (*on*/*off*), and (5) straw chopper balance status (*balanced*/*unbalanced*).

The choice of the *Exhaustive Search* method was possible due to the relatively small number of twelve features that were involved, as mentioned above. If more features were to be explored, it would be advisable to use *Sequential Forward/Backward Floating Search* for computational efficiency [29].

All the tasks of this stage were performed in the *MATLAB*^®^ programming environment using the *LIBSVM* library [57, 58].

### 3.4. SVM-Based Classification Stage

The classification stage took place once the previous processing stages had been performed. Among the huge variety of classifiers available, SVM classification was selected in this work because of its: (i) great generalization ability; (ii) low overtraining risk due to small datasets; and (iii) low computational load.

A different SVM-based classifier was employed for each of the five related classification problems, corresponding to the following five rotating component states of the agricultural harvester: (1) engine speed status (*high speed*/*low speed*); (2) threshing cylinder operating status (*on*/*off*); (3) threshing cylinder balance status (*balanced*/*unbalanced*); (4) straw chopper operating status (*on*/*off*); and (5) straw chopper balance status (*balanced*/*unbalanced*). The input of each classifier was the subset of features that led to maximum cross-validation accuracy (Section 3.3). If more than one subset led to the maximum value, only one of them was selected for the sake of simplicity. Each classifier provided one of the two classes associated with the input feature vector as its output.

For each of the five classifiers, the *linear* kernel and the *radial basis function* (RBF), the *sigmoidal*, and the *third-order polynomial* nonlinear kernels were employed, providing a comparison between their accuracy. These SVM kernels were selected, because they are the most typical and widely used. The *C* parameter, involved in the SVM classification formulation, and the *γ* and *c*_0_ parameters, involved in the kernel, were optimized by conducting an exponential grid-search on these parameters [59]. The parameters that led to the highest cross-validation accuracy were selected.

The *LIBSVM* toolbox [57, 58], running in the *MATLAB*^®^ programming environment, was once again employed for classification tasks.

### 3.5. Classifier Performance Evaluation Stage

The *leave-one-out* cross-validation accuracy (Section 2.2), for each of the five individual classification problems under consideration, was computed to assess the goodness of the proposed classifying system. These five cross-validation accuracies, as well as the overall mean cross-validation accuracy, were used as a measurement of the accuracy of the SVM-based estimation method for each of the five aforementioned harvester states.

## 4. Results

The experimental results of the feature selection and classifier performance evaluation stages are presented in this section.

### 4.1. Feature Selection

**Figure 7.**
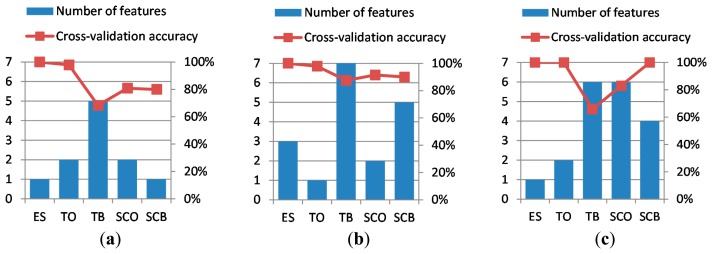
Number of features and cross-validation accuracy for each of the working conditions under consideration—(*ES*) engine speed, (*TO*) threshing cylinder operation, (*TB*) threshing cylinder balance, (*SCO*) straw chopper operation, and (*SCB*) straw chopper balance—using the accelerometer channel corresponding to (**a**) the transverse *X* axis; (**b**) the longitudinal *Y* axis; and (**c**) the vertical *Z* axis.

The selection of the best features, following the methods explained in Section 3.3, led to the best cross-validation accuracies and best particular chosen features shown in Table 1. It can be appreciated that, in all cases, the required number of features is lower than or equal to seven and that the mean cross-validation accuracy is above 85%, for all the three axes of the accelerometer. The best cross-validation accuracies and the number of features needed to achieve these are depicted in Figure 7.

### 4.2. SVM Classifier Performance Evaluation

**Figure 8.**
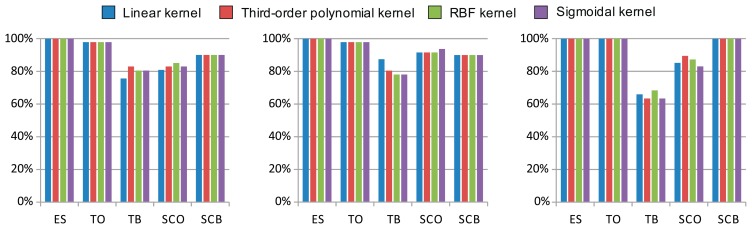
Cross-validation accuracy for each kernel under the following working conditions—(*ES*) engine speed, (*TO*) threshing cylinder operation, (*TB*) threshing cylinder balance, (*SCO*) straw chopper operation, and (*SCB*) straw chopper balance—using the accelerometer channel corresponding to (**a**) the transverse *X* axis; (**b**) the longitudinal *Y* axis; and (**c**) the vertical *Z* axis.

The results of the linear and nonlinear SVM classifier optimization, showing the best cross-validation accuracies and the related optimal parameters, are shown in Table 2. The previously selected features, highlighted in bold in Table 1, were used as input to the SVM classifier. Note that the nonlinear kernels did not outperform the linear kernel in most cases. Even in those cases where the accuracy was improved, only slight differences never over 10% were observed. It therefore appears that the use of linear SVM classification is sufficient to solve the problem. A comparison of kernel cross-validation accuracy is also provided in Figure 8.

## 5. Discussion

This article investigates a method of estimating the status of various rotating components in agro-industrial machinery by processing vibration signals acquired from a single point of the machine structure. It offers three major findings.

The first finding of this article is that it is possible to accurately estimate the status of some rotating components in agro-industrial machinery by processing the vibration signal acquired from a single point on the machine. Moreover, the accelerometer sensor does not need to be placed very close to the rotating components, which makes the acquisition stage simple and non-intrusive. The results presented above reveal the potential of this method to estimate the status of distant components by processing vibration signals from a unique sensor located at a fixed position, midway along the harvester chassis (Figure 5), because a mean cross-validation accuracy higher than 85% was obtained. Previous work in the scientific literature has only analyzed isolated mechanical components, using one accelerometer for each isolated component [19, 21, 56]. It is worth noting that, to the best of our knowledge, no previous articles have approached the problem of estimating the status of various mechanical components from a unique vibration signal.

The second finding of this article is that the vibration signal can be acquired with a uniaxial accelerometer, the orientation of which has no significant effect on classification accuracy. The comparison of the results of cross-validation accuracy along the three accelerometer axes (Table 2) supports this conclusion. The higher differences observable in Table 2 for the threshing cylinder balance status shows differences of around 20%. However, almost no differences in accuracy were appreciated for the rest of the states, which were lower than 10% in all cases. Although vibrations are usually generated in a specific direction, the results obtained here suggest that the machine structure spreads them along all of the axes, making the use of an arbitrary axis for their detection possible.

The third finding of this article is that, when using an SVM classifier, an 85% mean cross-validation accuracy can be reached, which only requires a maximum of seven features as its input, with no significant noticeable improvements from using nonlinear rather than linear kernels. Reviewing the results, a mean cross-validation accuracy greater than 85% was achieved, irrespective of the selected accelerometer axis. Analyzing the individual cross-validation accuracy obtained for each rotating component, the suitability of the SVM classifier for estimating each separate machinery status is evident. On the one hand, the rotating component status with the best cross-validation accuracy was the engine speed, with a cross-validation accuracy of 100% in all cases (Table 2). On the other hand, the worst cross-validation accuracy was obtained for threshing cylinder balance status, for which the cross-validation accuracy was between 63.41% and 87.49% (Table 2). A visual analysis of the vibration signal spectrum, revealed differences when the engine speed varied between high and low speed, while there were no visible changes in the signal spectrum when the threshing cylinder was either balanced or unbalanced. These results show that the proposed SVM classifier is able to classify the status of rotating machinery to a high degree of accuracy when the difference between the spectrum signals is noticeable, such as in the case of the engine speed status. They also show that it can obtain an acceptable cross-validation accuracy for rotating components when there is no visible difference between the spectrum signals, such as the threshing cylinder balance status. Comparing the fault detection accuracy in the present article against the results of Samanta *et al.* [56], who proposed an ANN-based classifier for the fault diagnostics of roller bearings based on data from several vibration signals and extracting only five time-domain features, this study has reported poorer results. Nevertheless, these differences can be justified by taking into account that in Samanta's article five vibration signals from different locations of a unique component were processed and because they were clean, as they came from the isolated mechanical component under analysis. Nevertheless, only one accelerometer sensor is employed in the present article to detect five states of three different rotating components and, furthermore, the vibration signal that is processed contains the superposed signals coming from the three components under analysis as well as from the other components of the machine. As can easily be understood, the present article approaches a much harder problem.

The major strength of the system proposed in this article is the simplicity of the data acquisition stage, employing only one sensor located at a single point on the machine for measuring the vibration signals. It is worth highlighting an article from Sugumaran *et al.* [21], who proposed an SVM-based classifier for the fault diagnostics of a unique roller bearing employing only one vibration signal. Our study, even though similar to Sugumaran's, is wider in the sense of trying to assess several machinery rotating components at once instead of just one. Furthermore, the present article contemplates the detection of further machine states and not only fault diagnostics.

Another strength of this article is that the proposed estimation method only needs seven features, at most, as the classifier input, yielding a simple SVM classifier with a low associated computational load. Moreover, the results showed no great differences in relation to the SVM kernel that was employed, which highlights that a simpler linear SVM classifier is sufficient to achieve good classification accuracy.

Nevertheless, there is a limitation to this work, which should be taken into account before implementing the proposed estimation method. This limitation is related to the data acquisition process performed in this article to validate the proposed SVM-based system. The vibration signals were acquired with the harvester wheels stopped to facilitate the acquisition procedure. If the proposed estimation method were to be used when the monitored machine is in motion, low-frequency interference signals could appear. However, these signals are not expected to cause problems, because the frequencies of interest in the rotating components of these machines will almost certainly be much higher than the interference frequencies.

The main application fields of the proposed SVM-based system are machinery monitoring and predictive maintenance. In relation to machinery monitoring, this system could be used for detecting the operating status of particular mechanical components, simplifying the wiring and reducing the number of sensors that are required. In relation to predictive maintenance, the results suggest that further progress may lead to fast and low-cost machinery inspections, thereby avoiding many mechanical faults and replacing expensive, time-consuming inspections that are frequently required nowadays.

A mixture of both conventional vibration signal analysis features, such as frequency- domain [4, 6, 19, 21, 27, 34] and time-domain [54–56] based features, and other unconventional features, such as nonlinear features [33, 37, 39], has been used in this article. The good classification accuracy levels, obtained for example with the *Central Tendency Measurement* feature when estimating the engine speed status (Table 1), highlights the usefulness of these unconventional features in the analysis of vibration signals for predictive maintenance. Nevertheless, regarding the straw chopper unbalance detection, the nonlinear features seem to be of little use. A future line for further research is opened by employing other unconventional features in vibration analysis for predictive maintenance.

Furthermore, this article opens a new future line of research by extending the system that is proposed in this paper to the use of more than one accelerometer located at different points on the machine. It is expected that the processing of all those signals together could enable the estimation of an even higher number of machine states and could also improve the accuracy of the estimation.

## 6. Conclusions

The results obtained in this study have provided evidence that (i) accurate estimation of the status of various rotating components in agro-industrial machinery is possible by processing the vibration signal acquired from a single point on the machine structure; (ii) the vibration signal can be acquired with a uniaxial accelerometer, the orientation of which does not significantly affect the classification accuracy; and, (iii) when using an SVM classifier, an 85% mean cross-validation accuracy can be reached, which only requires a maximum of seven features as its input, and no significant improvements are noted between the use of either nonlinear or linear kernels. Follow up research may lead to a simplification of the wiring and a reduction in the number of sensors required in machinery monitoring, as well as to fast and low cost machinery inspections in predictive maintenance.

## Figures and Tables

**Table 1.** Feature selection results for each of the three axes acquired by the triaxial accelerometer. The first row (number of features) shows the optimal number required to achieve the best cross-validation accuracy. The second row (best feature subset) shows all of the concrete feature subsets, giving the highest cross-validation accuracy as a list of numbers the legend of which corresponds to the list provided in Section 3.3. The subset employed for the subsequent classifier performance evaluation stage appears in bold. Each column corresponds to each of the rotating component classification problem under consideration.

		**Classification Problem**				
		***Engine Speed***	***Threshing Cylinder Operation***	***Threshing Cylinder Balance***	***Straw Chopper Operation***	***Straw Chopper Balance***
**Transverse X axis of the accelerometer**	**Number of features**	1	2	5	2	1
	**Best feature subset(s)**	**{9}**	**{10, 11}**	**{4, 6, 7, 9, 11}**	**{2, 8}**;{2, 5}	**5**
	**Cross-validation accuracy**	100%	97.87%	68.29%	80.85%	80%
	**Mean CVA**	85.40%				
**Longitudinal Y axis of the accelerometer**	**Number of features**	3	1	7	2	5
	**Best feature subset(s)**	**{6, 7, 10}**; {5, 7, 9}; {5, 6, 10}; {1, 6, 10}	**{5}**	**{1, 2, 7, 8, 9, 11, 12}**; {1, 2, 4, 7, 8, 11, 12}; {1, 2, 4, 6, 7, 11, 12}; {1, 2, 3, 5, 7, 8, 11}	**{4, 11}**	**{1, 3, 5, 7, 12}**
	**Cross-validation accuracy**	100%	97.87%	87.49%	91.49%	90%
	**Mean CVA**	93.37%				
**Vertical Z axis of the accelerometer**	**Number of features**	1	2	6	6	4
	**Best feature subset(s)**	**{9}**	**{2, 11}**;{2, 3}	**{2, 3, 5, 6, 8, 11}**	**{1, 4, 5, 7, 8, 10}**	**{1, 2, 7, 10}**
	**Cross-validation accuracy**	100%	100%	65.85%	82.98%	100%
	**Mean CVA**	89.77%				

**Table 2.** Performance results for each of the three axes acquired by the triaxial accelerometer, comparing the different SVM kernels, and showing both the optimized parameters (*C*, γ, *c_0_*) and the best cross-validation accuracy (CVA). The best result for each classification problem appears in bold.

			**Classification Problem**				
			***Engine Speed***	***Threshing Cylinder Operation***	***Threshing Cylinder Balance***	***Straw Chopper Operation***	***Straw Chopper Balance***
**Transverse *X* axis of the accelerometer**	**Linear kernel**	CVA	**100%**	**97.87%**	75.61%	80.85%	**90.00%**
		C	1	1	6	1	1.2
	**Third-order polynomial kernel**	CVA	**100%**	**97.87%**	**82.93%**	82.98%	**90.00%**
		C	0.03	32768	8192	2048	0.03
		γ	8	0.125	0.125	32	8
		c_0_	0.03	0.03	0.5	0.5	0.03
	**RBF kernel**	CVA	**100%**	**97.87%**	80.49%	**85.10%**	**90.00%**
		C	0.125	512	32	32	0.5
		γ	2	0.125	2	8	2
	**Sigmoidal kernel**	CVA	**100%**	**97.87%**	80.49%	82.98%	**90.00%**
		C	2	2048	2048	8	2
		γ	0.5	0.125	0.125	8	2
		c_0_	0.03	0.03	0.03	0.5	0.03
**Longitudinal *Y* axis of the accelerometer**	**Linear kernel**	CVA	**100%**	**97.87%**	**87.49%**	91.49%	**90.00%**
		C	1	1	1	1	1
	**Third-order polynomial kernel**	CVA	**100%**	**97.87%**	80.49%	91.49%	**90.00%**
		C	8192	0.03	8192	0.03	2048
		γ	0.125	8	0.002	8	0.125
		c_0_	0.03	0.03	8	0.03	0.03
	**RBF kernel**	CVA	**100%**	**97.87%**	78.05%	91.49%	**90.00%**
		C	0.5	0.5	2	2	2
		γ	8	8	0.5	2	2
	**Sigmoidal kernel**	CVA	**100%**	**97.87%**	78.05%	**93.62%**	**90.00%**
		C	2	32	512	8192	8
		γ	0.5	0.125	0.008	0.125	0.5
		c_0_	0.03	0.03	0.03	0.125	0.03
**Vertical *Z* axis of the accelerometer**	**Linear kernel**	CVA	**100%**	**100%**	65.85%	85.10%	**100%**
		C	1	1	1	430	1
	**Third-order polynomial kernel**	CVA	**100%**	**100%**	63.41%	**89.36%**	**100%**
		C	8192	512	2048	8192	0.03
		γ	0.125	0.5	0.125	0.03	8
		c_0_	0.03	0.03	0.5	2	0.03
	**RBF kernel**	CVA	**100%**	**100%**	**68.29%**	87.23%	**100%**
		C	0.125	32	2	8192	2
		γ	2	0.5	32	0.03	0.5
	**Sigmoidal kernel**	CVA	**100%**	**100%**	63.41%	82.97%	**100%**
		C	2	128	512	32	8
		γ	0.5	0.125	0.5	0.125	0.5
		c_0_	0.03	0.03	0.125	0.03	0.03
